# Subepithelial white deposition in the cornea associated with silicone oil and surgical management: a case report

**DOI:** 10.3389/fmed.2023.1147767

**Published:** 2023-06-09

**Authors:** Yuting Shao, Xin Liu, Yiyang Shu, Xiao Lv, Yanlong Bi

**Affiliations:** ^1^Department of Ophthalmology, Tongji Hospital, School of Medicine, Tongji University, Shanghai, China; ^2^Department of Ophthalmology, Guizhou Provincial People's Hospital, Guiyang, Guizhou, China; ^3^Tongji Eye Institute, Tongji University, Shanghai, China

**Keywords:** keratopathy, silicone oil, eccentric thickening, amniotic membrane transplantation, anterior segment optical coherence tomography

## Abstract

A 36-year-old patient presented with a complaint of an extensive “white scar” in his right eye without pain after silicone oil presence in the vitreous cavity for 12 years. Slit-lamp microscopy revealed extensive corneal leukoplakia and mild limbus neovascularization. Anterior segment optical coherence tomography revealed marked eccentric thickening of the subepithelium and normal thickness of the stroma. We proceeded with silicone oil removal and intraocular and anterior chamber lavage at first, followed by epithelial lesion excision combined with amniotic membrane transplantation 3 months later. The patient was satisfied with the clear cornea appearance.

## 1. Introduction

Since silicone oil (SO) was first introduced by Cibis in the 1960's, it has played an irreplaceable role as a long-term vitreous substitute in managing eyes with complicated vitreoretinal diseases after pars plana vitrectomy (1, 2). Although SO has several advantages compared with other tamponades, the incidence of multiple complications, such as intraocular inflammation, keratopathy, cataract, intraocular hypertension, glaucoma, peri-oil fibrosis, epiretinal membrane, and retinal toxicity, increases with the extension of filling time (2–5). Keratopathy associated with SO has been reported to mainly include band-shaped keratopathy (BSK) and bullous keratopathy, for which the prevalence varies from 7 to 29% (6, 7). BSK is characterized by grayish-white opacities deposited in the superficial corneal layers. The chelation of calcium with topical EDTA is the most common surgical intervention (8). Bullous keratopathy presents with corneal edema and extensive or local scarring due to corneal endothelial decompensation. Penetrating keratoplasty or selective posterior lamellar procedures are candidate surgical options only if the patient has good visual potential (7, 9).

In this study, we report a special case of a young man with a history of SO implantation for 12 years who presented with an extensive eccentric corneal “white scar” and obtained a significantly clearer cornea with two-stage surgery.

## 2. Case report

**Figure 1:**
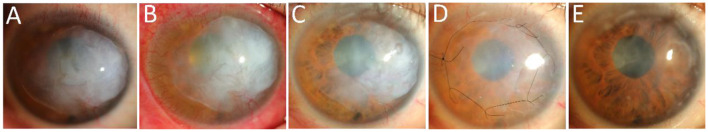
Changes in the clinical appearance of the right eye in slit-lamp microscopy. **(A)** Appearance of the patient's right eye at initial diagnosis. **(B)** Appearance of the patient's right eye after SO removal and adequate intraocular and anterior chamber lavage. **(C)** Appearance of the right eye 2 months after the first-stage surgery. **(D)** Appearance of the right eye after epithelial lesion excision combined with amniotic membrane transplantation 3 months after the first-stage surgery was completed. **(E)** Appearance of the right eye 2 months after the second-stage surgery.

**Figure 2:**
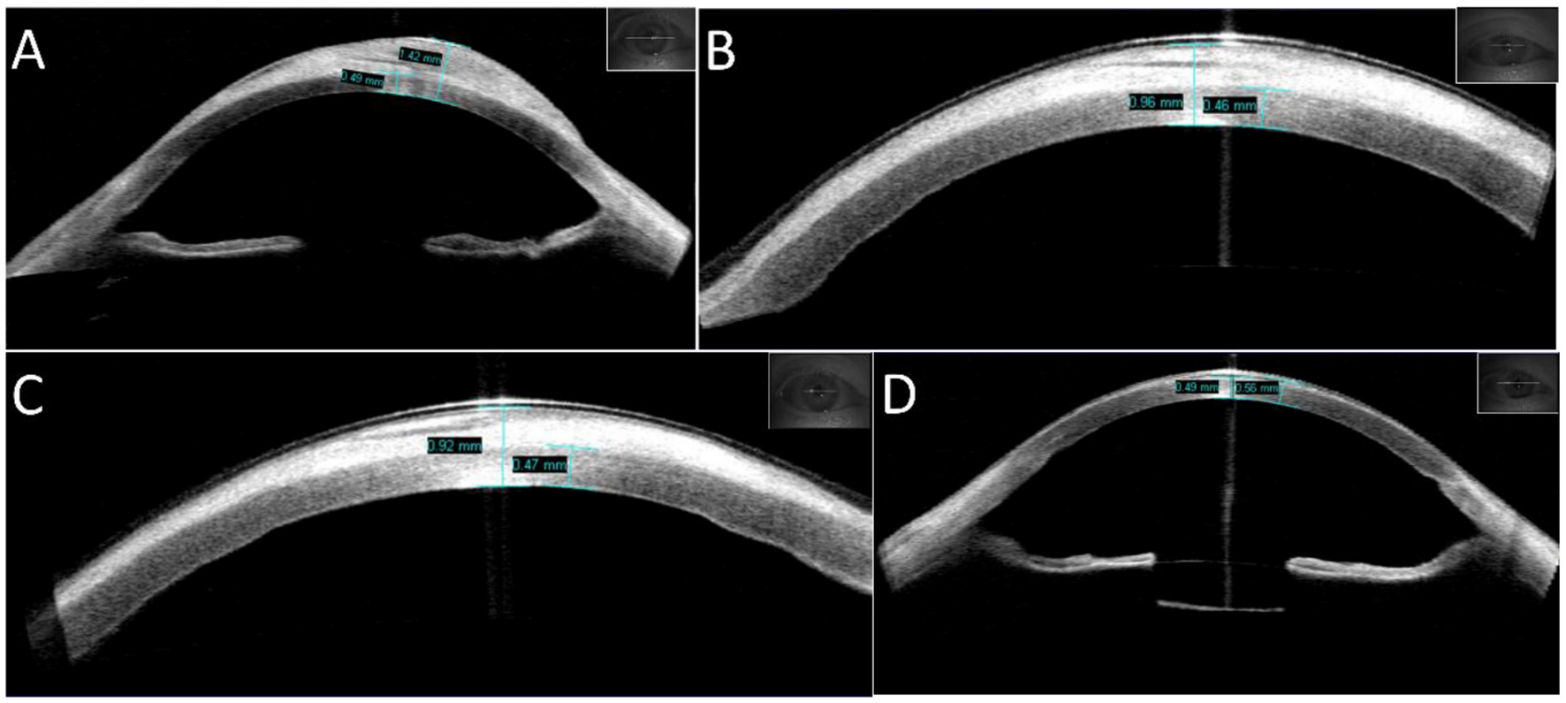
Changes in the appearance of the cornea measured using AS-OCT. **(A)** Appearance of the patient's right eye at initial diagnosis, with an eccentric thickness of the subepithelium (maximum thickness of cornea: 1.42 mm) and normal thickness of the stroma (0.49 mm). **(B)** Appearance of the patient's right eye 2 days after SO removal and adequate intraocular and anterior chamber lavage, with a smaller thickness of the cornea (0.96 mm). **(C)** Appearance of the right eye 2 months after the first-stage surgery, with a further decrease in the thickness of the cornea (0.92 mm). **(D)** Appearance of the right eye 2 months after second-stage surgery, with normal thickness of the cornea (0.55 mm). The lens capsular bag is visible.

A 36-year-old man presented to the ophthalmology clinic with complaints of extensive “white scarring” in the right eye without pain. He had a history of unexplained fundus hemorrhage and underwent vitrectomy and extracapsular lens extraction with SO left in the vitreous cavity of the right eye for 12 years. He had no eye trauma or other basic diseases. On examination, his right eye had no light perception and the intraocular pressure (IOP) was 40 mmHg. Extensive corneal leukoplakia, mild limbus neovascularization, and aphakia were found (Figure 1A) using slit-lamp microscopy. The fundus was invisible. A diagnosis of SO-associated keratopathy was given. Anterior segment optical coherence tomography (AS-OCT) further revealed a marked eccentric thickening of the subepithelium and normal thickness of the stroma (Figure 2A), inconsistent with previous silicone oil keratopathy cases of bullous keratopathy or BSK. For cosmetic and safety purposes, SO removal and adequate intraocular and anterior chamber lavage were conducted at the first stage. One percent prednisolone acetate, 0.3% levofloxacin, pranoprofen, and 0.1% sodium hyaluronate eye drops were administered four times a day under close observation. However, exudative retinal detachment was subsequently observed under B-scan ultrasound on the second postoperative day, but natural reattachment was observed 10 days later. The corneal leukoplakia had faded slightly (Figures 1B, C, 2B, C) at the 2-month follow-up. Epithelial lesion excision combined with amniotic membrane transplantation (Figure 1D) was performed 3 months after the first-stage surgery. Levofloxacin (0.3%), sodium hyaluronate (0.1%), recombinant bovine basic fibroblast growth factor eye drops (Essex Bio-Technology GmbH, China), and a bandage contact lens (Bausch & Lomb Incorporated, USA) were administered to accelerate epithelial healing and prevent infection. A significantly clearer cornea (Figures 1E, 2D) was observed at the next 2-month follow-up. Due to high intraocular pressure for years, visual acuity was limited to no light perception for optic nerve atrophy, and antiglaucoma medications were continuously used to control IOP. The young patient was satisfied with the treatment effect.

## 3. Discussion

Keratopathy is one of the most frequently observed complications associated with SO tamponade (2, 4). The universal clinical diagnosis of SO-associated keratopathy under slit-lamp microscopy mainly includes bullous keratopathy and BSK, for which treatments have been recognized by specialists (7, 8).

The exact mechanism of SO-induced pathological changes in the cornea remains unknown. For BSK, large SO bubbles or numerous emulsified SO droplets block the transport of nutrients from the aqueous to the cornea, causing metabolic disorder of calcium salt deposits in Bowman's layer and superficial stroma (9–12). For bullous keratopathy, persistent contact of SO with the endothelium causes progressive damage until decompensation (9). In this case, other manifestations, including uneven white opacification of the cornea under slit-lamp microscopy as well as eccentric thickness of the subepithelium and normal thickness of the stroma, as observed in AS-OCT, were present. Although we did not calculate the endothelial cell density, the cornea was clear and transparent at the last follow-up, indicating that endothelial decompensation did not occur at first. The manifestations mentioned above remain to be explained. We speculate that these special manifestations are attributed to the tiny size and migration of SO into the cornea.

Chan et al. found that over 65% of emulsified SO droplets in clinical samples had a median size between 1.1 and 1.9 μm, which cannot be seen by slit-lamp microscopy (13). Hence, tiny emulsified SO droplets can pass through anatomically compromised areas, such as zonules, locally damaged endothelial cells, or Descemet membranes, and then deposit under the epithelium. Le et al. also observed hyperreflective deposits with various shapes in both the posterior and anterior stroma using *in vivo* laser scanning confocal microscopy (IVCM), along with Langerhans cells infiltrating around stromal deposits (14). The infiltration of Langerhans cells and the appearance of neovascularization may be due to a rejection reaction to emulsified SO droplets. Mansour et al. also used AS-OCT to detect a case of SO-associated keratopathy and revealed a marked thickening of the stroma with diffuse round empty spaces between fibers. The authors speculated that this corresponds to intrastromal SO droplets (15). We share a similar opinion whereby subepithelial deposits in this case could originate from the migrated, emulsified SO. Moreover, the whitish color of the cornea is similar to that of emulsified SO. Therefore, the uneven white deposits under the subepithelium in the cornea may be caused by the migration of numerous tiny SO droplets from locally damaged tissue. It is neither related to endothelium malfunction nor similar to metabolic disorder in BSK.

Lee et al. noted that SO removed prior to or during PK is beneficial to the graft's long-term success (16). In this case, the first stage surgery included SO removal with vitreous and anterior chamber lavage. Subsequently, corneal leukoplakia faded slightly in the following 2 months probably due to the relief of insult from SO. However, the SO removal procedure still requires vigilance, as, in addition to the possibility of recurrent retinal detachment, acute corneal decompensation may also occur (17). In eyes with acute corneal decompensation, SO usually completely fills the anterior chamber, acting as a physical barrier that prevents access of the aqueous to the stroma, forming the illusion of a healthy cornea under slit-lamp microscopy. However, according to specular microscopy or IVCM, significantly decreased endothelial cell density and hexagonal cells is observed (17, 18). In this case, we were unable to evaluate the endothelial cells through the above examination due to the white deposits; however, we identified no large SO bubbles in the anterior chamber due to the existence of a capsular bag, as observed in AS-OCT (Figure 2D). In addition, we also learned about the patient's lifestyle. The patient was used to sleeping on his side and usually worked in a sitting or standing position. All these positions led to peripheral contact between SO droplets and the corneal endothelium rather than central contact, which caused greater damage to the cornea. In addition, the 36-year-old patient was younger than the man in a previous report, and his endothelium had a greater capacity to withstand damage (13). Therefore, we decided to remove the SO at the first stage after speculating on his fine corneal endothelium.

Notably, this patient did not present with pain, and his purpose was corneal cosmetology. This also suggests that the disease differs from the common keratopathy associated with SO. Wang et al. reported a case of a 30-year-old man with SO filling for over 12 years. Similar but more serious corneal opacification with severe pain presented. B-scan ultrasound and computed tomography scanning showed retinal detachment and calcification of the eyeball wall. Evisceration was finally performed to relieve the painful, blind eye (19). Hence, the patient's symptoms and purpose are important clues for our diagnosis and treatment. The limitation of this study is that we did not conduct a composition analysis of the white deposits removed during surgery. In future studies, we will collect more cases to study and reveal the pathophysiological mechanisms of SO-associated keratopathy.

## 4. Conclusion

In conclusion, we report a special keratopathy associated with SO that is inconsistent with previously reported cases. We emphasize that the size and migration of SO in the anterior chamber should be carefully identified. The evaluation of the characteristics of the cornea with the help of AS-OCT contributes to selecting surgical interventions. Cooperation between cornea and vitreoretinal specialists is also quite important. Otherwise, cornea specialists should learn cross-domain knowledge to avoid inadequate or incorrect treatment.

## Data availability statement

The original contributions presented in the study are included in the article/supplementary material, further inquiries can be directed to the corresponding author.

## Ethics statement

Written informed consent was obtained from the individual(s) for the publication of any potentially identifiable images or data included in this article.

## Author contributions

YB managed the patient and created the assessment and plan. XLi and XLv contributed to data acquisition. YSha drafted the manuscript. YShu performed the examinations for the patient. All the authors read and reviewed the manuscript. All authors contributed to the article and approved the submitted version.
